# Untangling Caregiving Role From Parent Gender in Coparenting Research: Insights From Gay Two-Father Families

**DOI:** 10.3389/fpsyg.2022.863050

**Published:** 2022-03-16

**Authors:** Nicola Carone, Vittorio Lingiardi

**Affiliations:** ^1^Department of Brain and Behavioral Sciences, University of Pavia, Pavia, Italy; ^2^Department of Dynamic and Clinical Psychology, and Health Studies, Sapienza University of Rome, Rome, Italy

**Keywords:** coparenting, caregiving roles, parent gender, gay fathers, parenting, sexual minority parent families, parent involvement in childcare, unpaid labor

## Introduction

Research suggests that heterosexual fathers display similar parenting behaviors as heterosexual mothers, and have an analogous influence on children's development (Fagan et al., 2014; Volling and Cabrera, 2019; Volling and Palkovitz, 2021). However, claims that heterosexual fathers make a unique contribution to children's development (Jeynes, 2016) persist, often attributed to evolved differences between males and females (Paquette, 2004). Additionally, heterosexual mothers and fathers typically take on distinct coparenting roles, with mothers assuming more non-paid tasks (e.g., Yavorsky et al., 2015) and devoting two to three times as much time with their children, relative to fathers (Cabrera et al., 2018).

The increasing number of gay two-father families worldwide (Blake et al., 2017; Berkowitz, 2020; Carone et al., 2021) may allow us to expand our theoretical understanding of coparenting and child development within diverse family structures. Uniquely, gay two-father families involve two fathers and no mother, and both parents have a non-heterosexual orientation. Additionally, depending on whether surrogacy or adoption was used, either one or two of the fathers is biologically unrelated to their child, respectively. Accordingly, research with gay two-father families promises novel and significant insight into coparenting dynamics.

To date, with few exceptions (e.g., Farr and Patterson, 2013; Tornello et al., 2015; Carone et al., 2017; Farr et al., 2019; van Rijn-van Gelderen et al., 2020), coparenting research has focused on heterosexual two-parent families with biological children (Feinberg, 2003; McHale, 2011). In such families, caregiving roles are generally defined according to parent gender. Potential variations in coparenting according to parents' sexual orientation and parent–child (non-)biological relatedness (and the interaction between these factors) have not been addressed. Since research has documented the unique predictive power of coparenting for child adjustment across developmental stages (Teubert and Pinquart, 2010), it seems fundamental to examine the extent to which coparenting is influenced by parent gender and caregiving role, while accounting for the contribution of parent sexual orientation and biological (non-)relatedness.

This opinion article presents an overview of recent findings relating to gay fathers (through adoption and surrogacy) to differentiate the effects of caregiving role and parent gender, identifying the unique and joint contributions of these factors to coparenting behaviors. Given that less research about coparenting has focused on gay fathers than lesbian mothers, where relevant, studies with the latter group are also included.

## The (Ir)relevance of Parent Gender for Coparenting

Heterosexual women's participation in the labor force, and their associated political and social achievements, have increased over recent decades. However, in heterosexual two-parent families, these gains have not translated into a more egalitarian allocation of household labor (Lachance-Grzela and Bouchard, 2010). This is true also among highly educated couples, with both partners employed full-time (Cabrera et al., 2018). This contrasts the relative resource theory (Blood and Wolfe, 1960), which predicts that the division of parenting tasks results mainly from differences in parental resources. Another explanation for this pattern relies on the gender roles and gender ideology, which are embedded societally and internalized and enacted during coparenting (Lachance-Grzela and Bouchard, 2010).

As gay fathers are less susceptible to pressure to conform to gender roles, they may be more likely than heterosexual couples to contribute equally to coparenting. A U.S.-based study comparing 29 gay, 25 lesbian, and 50 heterosexual adoptive couples on coparenting practices with 3-year-old children showed that the gay and lesbian couples were more likely to share parenting tasks evenly than the heterosexual couples (Farr and Patterson, 2013). In the follow-up study in middle childhood, no differences in coparenting emerged across family types (Farr et al., 2019). However, a Canadian study with 92 adoptive gay fathers with children aged 1–9 years concluded that gender roles may predict overall involvement in childcare, as the fathers who reported higher femininity were most involved (Feugé et al., 2019).

This last result opens space for further reflecting on what “femininity” (and “masculinity”) stand for in coparenting. Said differently, it might be that the greater involvement of “more feminine” fathers does not have so much (or not only) to do with gender roles as it has to do with psychic identifications with (co)parenting functions experienced in their own family of origin? We are thinking here on Kentlyn's (2007) result that, for many gay and lesbian parents who did more household labor, it was like “coming back to the base”, that is to their internalized maternal function. This is not to perpetuate rigid stereotypes of femininity and masculinity and, consequently, what is expected from mothering and fathering. Rather, we aim at stimulating more reflections on the influence of internalized early relational experiences on the distribution of household labor as adults. In this vein, further research on the interaction between gender roles and identifications with parenting functions would shed light on a much less explored part of the coparenting story.

Looking at coparenting among heterosexual mothers and fathers, social structural theory (Eagly and Wood, 1999) offers an alternative explanation of differences between them, arguing that “the roles people occupy—which may be due to individual choice, sociocultural pressures, or biological potentials—lead them to develop psychological qualities and, in turn, behavior to fit those roles” (Katz-Wise et al., 2010, p. 2). From this perspective, biological differences between mothers and fathers (especially related to experiences of pregnancy, birth, and breastfeeding) may determine that heterosexual mothers spend more time engaging in childcare relative to heterosexual fathers. This view was supported by a study on task division involving lesbian mothers, showing that biological mothers tended to invest more time in childcare than non-biological mothers with children aged 3 months (Goldberg and Perry-Jenkins, 2007). Studies with older children of lesbian mothers, however, have produced mixed findings (e.g., Chan et al., 1998; Bos et al., 2007; Downing and Goldberg, 2011), suggesting that lesbian mothers may have a more flexible caregiving division that changes over time.

Among gay fathers through surrogacy, biological relatedness seems unrelated to levels of involvement in household and childcare tasks (Tornello et al., 2015; van Rijn-van Gelderen et al., 2020). However, it may matter for the conflictual dimension of coparenting, as an Italian study (Carone et al., 2017) found that non-biological fathers reported less undermining coparenting compared to biological fathers. This contradicts the theory of selection (Hamilton, 1964), which assumes that altruistic behavior is adaptive when it increases genetic fitness; thus, due to the economic, physical, and mental costs of raising a child, biological gay fathers should invest more in childcare relative to non-biological gay fathers.

A further variable to consider is parents' time spent outside the home: according to time-constraint theory (Artis and Pavalko, 2003), the parent who spends more hours at work and engaging in external activities will have less time to invest in household and childcare tasks. Indeed, Patterson et al. (2004) found that lesbian mothers spent the same number of hours in paid employment and were equally involved in childcare, while heterosexual fathers spent twice as many hours in paid employment as did their female partners, resulting in the mothers being more intensively involved in childcare.

The results with gay fathers have varied. A U.S. study with 335 gay fathers through different paths to parenthood found that the father who worked fewer hours in paid employment relative to their partner performed more of the household and childcare labor. Conversely, in Feugé et al.'s study of adoptive fathers (Feugé et al., 2019), no relation was found between parental involvement (including perceived involvement) and hours devoted to paid work.

By definition, heterosexual two-parent families and gay two-father families differ. We cannot ignore the function played by parent gender in organizing coparenting in heterosexual two-parent families, as a result of historical, socio-cultural, and political factors (Lachance-Grzela and Bouchard, 2010). On the other hand, studies on coparenting among gay fathers have not yet produced firm indications. Nonetheless, they invite us to “look beyond the hood” of parent gender to capture a broader array of factors that might influence coparenting behaviors and determine “how mothers and fathers are similar, different, complementary, or additive” (Cabrera et al., 2018, p. 3). Caregiving role might be one of those factors.

## Fathers as Primary Caregivers

Cultural shifts in norms of masculinity and femininity have encouraged a growth in the number of primary caregiving heterosexual fathers (i.e. “stay-at-home fathers”) (Solomon, 2014). These fathers are shifting away from traditional/hegemonic forms of masculinity and embracing more nurturing forms of fathering (Hunter et al., 2017). However, in their attempts to integrate into the “parenting space” that has traditionally been occupied by mothers, they are experiencing stigmatization (Coltrane et al., 2013) and “gender discrepancy strain” (Pleck, 1995). Future research should consider whether these challenges are reducing the quality of their coparenting.

Although heterosexual fathers are increasingly embracing the role of primary caregiver and heterosexual mothers are increasingly taking on the role of primary earner (Schoppe-Sullivan and Fagan, 2020), in the majority of heterosexual two-parent families, mothers still remain more engaged in childcare. In gay two-father families, the distinction between primary and secondary caregiver is not always marked; as a result, researchers must sometimes randomly assign the primary caregiving role to one father (van Rijn-van Gelderen et al., 2020) or label both fathers primary caregivers (Ellis-Davies et al., 2022). Thus, gay two-father families are encouraging a redefinition of caregiving roles, as parental gender is no longer the defining criterion.

Emerging attachment research with gay two-father families (Feugé et al., 2019; Carone et al., 2020; Ellis-Davies et al., 2022) has contributed promising and novel insights that can be extended to coparenting. In heterosexual two-parent families, Bretherton (2010) found that mothers and fathers differentiate their attachment roles such that mothers primarily address safe haven needs whereas fathers primarily support secure exploration. Kerns et al. (2015) further showed the different—somewhat complementary—roles played by mothers and fathers as attachment figures.

Nevertheless, one key question arising from such attachment research (e.g., Bretherton, 2010; Kerns et al., 2015) is whether—and to what extent—children's tendency to use mothers as safe havens and fathers as secure bases is due to a conflation between caregiving role and parental gender. To address this, we studied 33 gay two-father families through surrogacy and 37 lesbian two-mother families through donor insemination, with children aged 6–12 years (Carone et al., 2020). Our aim was to investigate how children used their parents to fulfill safe haven and secure base needs, comparing family groups in which the parents were of the same gender, only one parent was biologically (non-)related to their child, and caregiving roles were likely to be shared equally.

The results indicated that, irrespective of family type, children used the primary caregiver more as a safe haven and the secondary caregiver more as a secure base, though they reported high levels of both types of support from both parents. This suggests that, when children's attachment needs cannot be obviously addressed on the basis of parent gender, caregiving roles may explain variations in child–parent interactions. From a psychodynamic perspective, this implies that each parent, regardless of their gender, remains a fundamental attachment figure who transmits their internal model of relationships to their child through parenting behavior, partly independent of the other parent's actions (Fonagy et al., 1994; Steele et al., 1996). Through this mechanism, the child develops and maintains distinguishable mental representations of the expected relationship with each parent, and these representations combine into an integrated view of attachment relationships as the child matures (Fonagy et al., 1994).

Additional support for the relevance of caregiving role over parent gender comes from a recent neurobiological parenting study. Abraham et al. (2014) compared the brain activity of primary caregiving gay fathers through surrogacy with that of primary caregiving heterosexual mothers and secondary caregiving heterosexual fathers through unassisted conception, all of whom were first-time parents of infants. While the heterosexual mothers and heterosexual fathers showed heightened activity in brain areas associated with emotion processing and cognitive processing, respectively, gay fathers showed increased activity in both of these regions. This indicates that primary caregiving gay fathers may respond similarly to both heterosexual mothers and fathers and that, in turn, the caregiver role might be relevant to fathers' and mothers' parenting qualities. Future research should include secondary caregiving gay fathers, primary caregiving lesbian mothers, and secondary caregiving lesbian mothers to capture potential interactions between caregiving role and parent gender in brain area activation during parenting (Giannotti et al., 2022).

## Discussion

The prevailing coparenting model in heterosexual two-parent families positions fathers as “helpers” to mothers (Cabrera et al., 2014, 2018; Schoppe-Sullivan and Fagan, 2020). Most coparenting research considers the mother the primary caregiver (and thus the representative parent in the family), because mothers typically spend more time with children than do fathers (Cabrera et al., 2018). However, the exclusion of fathers from coparenting research on this basis, and the subsequent use of mothers' reports only, contradict evidence that the quality of the parent–child relationship is more important than the quantity of parental involvement (Pleck, 2010). It also lacks ecological validity, since it systematically obscures families' actual daily experience. Thus, if our goal is to understand the effects of coparenting and the parent–child relationship on child development, an exclusive focus on mothers risks overestimating their effect (Schoppe-Sullivan and Fagan, 2020).

Our overview of studies involving gay fathers warns against an assumed overlap of caregiving role and parent gender, and stresses the need to consider these factors independently, also in heterosexual two-parent families. Researchers should ask mothers and fathers how they manage caregiving responsibilities, instead of assuming a-priori gender-based divisions, as well as explore the coparenting model they have experienced and internalized during childhood as it may reflect in their coparenting relationships as adults. Additionally, they should explore children's perceptions of their parents' caregiving roles. As such perceptions result from parents' transmission of their internal model of parenting, socialization practices, and actual parenting behaviors, they may differ—to some degree—from parents' own perceptions. In a similar vein, future research should investigate whether coparenting behaviors in heterosexual two-parent families develop from the complementary caregiving roles adopted by mothers and fathers, rather than the differentiation between women and men, respectively. Finally, cross-cultural research is needed to verify whether culture also contributes to the overlap of parent gender and caregiving role.

Further research might also compare families with a primary caregiver mother and a secondary caregiver father to families with a primary caregiver father and a secondary caregiver mother, to determine whether parent gender or parents' adoption of complementary roles explains different coparenting behaviors. Such a comparison could illuminate the unique and joint contributions of parent gender and caregiving role on child development through coparenting. In addition, coparenting research with parents of diverse genders and sexual orientations, as well as parents with biological (non-)relatedness to children, could contribute to either substantiating or disconfirming the notion that fathering and mothering are unique constructs (Fagan et al., 2014; Jeynes, 2016) and clarify whether—and under which circumstances—caregiving role and parent gender interact and, in turn, determine coparenting dynamics.

As a final remark, caregiving roles vary according to individual, couple, family, and contextual circumstances. For this reason, policies such as shared parental leave and flexible working have a significant impact on coparenting quality among heterosexual couples (e.g., Lidbeck and Bernhardsson, 2021), and should be widely promoted by governments and employers to support gender equality within families. During the COVID-19 lockdowns, heterosexual fathers' contributions to unpaid childcare increased, though heterosexual mothers still spent much more time on childcare relative to fathers (e.g., Andrew et al., 2020; Farré et al., 2020; Manzo and Minello, 2020). If homeworking continues into the long term and increasingly influences the (in)balance between heterosexual mothers and fathers in childcare, the untangling of caregiving role and parent gender will be fundamental for identifying more nuanced coparenting dynamics (e.g., similar parenting behaviors for both parents, more prevalent behaviors at a specific time, behaviors done by a specific parent, and behaviors that produce specific outcomes).

## Author Contributions

NC and VL substantially contributed to the conception of the manuscript. NC drafted the manuscript and VL revised it critically for important intellectual content. Both authors approved the manuscript for publication.

## Funding

This work was supported by the Ministry of University and Research under the call Progetti di Rilevante Interesse Nazionale (PRIN) 2017 (project number 2017XNYB9C).

## Author Disclaimer

The views expressed in the article are those of the authors and do not necessarily reflect the views of the Ministry of University and Research.

## Conflict of Interest

The authors declare that the research was conducted in the absence of any commercial or financial relationships that could be construed as a potential conflict of interest.

## Publisher's Note

All claims expressed in this article are solely those of the authors and do not necessarily represent those of their affiliated organizations, or those of the publisher, the editors and the reviewers. Any product that may be evaluated in this article, or claim that may be made by its manufacturer, is not guaranteed or endorsed by the publisher.
